# Size Uniformity of CsPbBr_3_ Perovskite Quantum Dots via Manganese-Doping

**DOI:** 10.3390/nano14151284

**Published:** 2024-07-30

**Authors:** Mi Zhang, Xue Han, Changgang Yang, Guofeng Zhang, Wenli Guo, Jialu Li, Zhihao Chen, Bin Li, Ruiyun Chen, Chengbing Qin, Jianyong Hu, Zhichun Yang, Ganying Zeng, Liantuan Xiao, Suotang Jia

**Affiliations:** 1 State Key Laboratory of Quantum Optics and Quantum Optics Devices, Institute of Laser Spectroscopy, Collaborative Innovation Center of Extreme Optics, Shanxi University, Taiyuan 030006, China; z15034657487@163.com (M.Z.); hanxuechn@163.com (X.H.); changgang.yang@sxu.edu.cn (C.Y.); guowenlisx@163.com (W.G.); jialu-li@outlook.com (J.L.); chenzhihaochn@163.com (Z.C.); libin@sxnu.edu.cn (B.L.); chenry@sxu.edu.cn (R.C.); chbqin@sxu.edu.cn (C.Q.); jyhu@sxu.edu.cn (J.H.); yangzhichun@sxu.edu.cn (Z.Y.); zengganying@sxu.edu.cn (G.Z.); tjia@sxu.edu.cn (S.J.); 2 College of Physics, Taiyuan University of Technology, Taiyuan 030006, China

**Keywords:** CsPbBr_3_ perovskite quantum dots, manganese-doping, size uniformity, single quantum-dot spectroscopy, narrow-linewidth

## Abstract

The achievement of size uniformity and monodispersity in perovskite quantum dots (QDs) requires the implementation of precise temperature control and the establishment of optimal reaction conditions. Nevertheless, the accurate control of a range of reaction variables represents a considerable challenge. This study addresses the aforementioned challenge by employing manganese (Mn) doping to achieve size uniformity in CsPbBr_3_ perovskite QDs without the necessity for the precise control of the reaction conditions. By optimizing the Mn:Pb ratio, it is possible to successfully dope CsPbBr_3_ QDs with the appropriate concentrations of Mn²⁺ and achieve a uniform size distribution. The spectroscopic measurements on single QDs indicate that the appropriate Mn²⁺ concentrations can result in a narrower spectral linewidth, a longer photoluminescence (PL) lifetime, and a reduced biexciton Auger recombination rate, thus positively affecting the PL properties. This study not only simplifies the size control of perovskite QDs but also demonstrates the potential of Mn-doped CsPbBr_3_ QDs for narrow-linewidth light-emitting diode applications.

## 1. Introduction

Cesium lead halide (CsPbX_3_, where X = Cl, Br, or I) perovskite quantum dots (QDs) display a range of advantageous properties, including high photoluminescence (PL) quantum yields, tunable PL spectra, high defect tolerance, and large carrier diffusion lengths [1,2,3,4]. These excellent properties have led to CsPbX_3_ perovskite QDs having considerable potential in a wide range of applications, including light-emitting diodes (LEDs) [5,6], photovoltaics [7], lasers [8,9], photodetectors [10,11], and quantum light sources [12]. Nevertheless, the properties and applications of nanoscale perovskite QDs are strongly dependent on their size uniformity and monodispersity. As the sizes of the QDs are smaller than their Bohr diameters, the energy levels are quantized due to quantum confinement effects. As the size of the QD decreases, the confinement of the carriers becomes stronger, leading to an increase in the energy spacing between the discrete energy levels [13,14]. Consequently, the optical and electronic properties of QDs are highly sensitive to their sizes. The non-uniformity of the size of QDs typically leads to the broadening of the PL spectra, which in turn affects the performance of QD-based devices [15,16,17,18]. Consequently, size uniformity is of paramount importance in determining the performance, stability, and controllability of QD-based applications. This ensures consistency in optical, electronic, and assembly properties, thereby improving the functionality and reliability of devices in a range of fields. Nevertheless, the production of perovskite QDs with high size uniformity using colloidal methods remains a significant challenge.

A variety of synthesis methods and strategies have been employed to achieve size uniformity and monodispersity in perovskite QDs. For example, precursor stoichiometry control has been used to precisely tune precursor ratios to influence nucleation and growth kinetics, resulting in uniform perovskite QD sizes [19,20]. The precise control of temperature and reaction time has been employed to regulate synthesis conditions, resulting in the formation of smaller, more uniform perovskite QDs [21,22]. Furthermore, solvent engineering has been utilized to optimize solubility and growth kinetics, thereby improving size control [23,24]. The use of ligands such as oleic acid or oleylamine in surfactant engineering has been employed to stabilize the perovskite QDs and enhance size uniformity [25,26]. However, maintaining precise temperature control and appropriate reaction conditions can be challenging, resulting in variations in perovskite QD size and uniformity.

In this study, we demonstrate the successful achievement of the size uniformity and monodispersity of CsPbBr_3_ perovskite QDs through the incorporation of manganese (Mn) doping. This method does not necessitate the precise control of various reaction conditions for the synthesis of CsPbBr_3_ perovskite QDs. It has been demonstrated that Mn doping in perovskite QDs can stabilize the crystal structure, effectively reduce the defect density, and improve the lattice ordering [26]. By optimizing the Mn:Pb ratio in the precursor, it was possible to successfully introduce appropriate concentrations of Mn^2+^ into the CsPbBr_3_ perovskite QDs. The introduction of Mn^2+^ greatly optimizes the size distribution of CsPbBr_3_ perovskite QDs. Single QD spectroscopy measurements demonstrate that the incorporation of Mn^2+^ at appropriate concentrations has a positive effect on the PL properties of CsPbBr_3_ perovskite QDs.

## 2. Materials and Methods

All materials were purchased from Aladdin and used as received without further purification. The materials used in the experiments include cesium carbonate (Cs_2_CO_3_, 99.9%, metals basis), lead bromide (PbBr_2_, 99.0%), zinc bromide (ZnBr_2_, 99.9%, metals basis), manganese (II) bromide (MnBr_2_, 98.0%), oleylamine (OAm, C18:80–90%), oleic acid (OA, AR), 1-octadecene (ODE, >90.0%(GC)), and hexane (>99%(GC)).

In this study, CsPbBr_3_ perovskite QDs were synthesized by suitably modifying the previously reported hot injection synthesis method [19]. Stoichiometric mixtures of different ratios of PbBr_2_ and MnBr_2_ were used for the synthesis of Mn-doped CsPbBr_3_ perovskite QDs with different doping concentrations. The Cs precursor solution was prepared by reacting 0.25 g of Cs_2_CO_3_ powder with 0.9 mL of OA and 9 mL of ODE in a 50 mL three-necked flask. This reaction was conducted at 150 °C under a nitrogen (N_2_) atmosphere. A separate solution was then prepared by mixing 75 mg of PbBr_2_, together with ZnBr_2_ (acting as a Br^−^ supplementation source) and MnBr_2_ (acting as a dopant) with 5 mL of ODE, 2 mL of OA, and 2 mL of OAm in a separate 50 mL round-bottomed three-necked flask; the mass and molar ratios of PbBr_2_, MnBr_2_ and ZnBr_2_ at different doping concentrations are given in Table S1. The aforementioned mixture was maintained at 120 °C under an N_2_ atmosphere for one hour to ensure thorough mixing and solubilization. Once the requisite temperature was reached, 0.4 mL of the previously prepared Cs precursor solution was rapidly injected into the flask containing the PbBr_2_ mixture. This injection initiated the nucleation and subsequent growth of the CsPbBr_3_ perovskite QDs. To promptly terminate the reaction, the flask was immersed in an ice bath after 5 s. After the reaction, the CsPbBr_3_ perovskite QDs were separated from the reaction mixture by high-speed centrifugation. Methyl acetate was used as an antisolvent to precipitate the perovskite QDs, which were then redispersed in hexane. Unreacted salts and impurities were removed as precipitates through centrifugation at 4000 rpm, and the supernatant, containing the dispersed perovskite QDs, was collected for further use. During the synthesis of the Mn-doped QDs, ZnBr_2_ was used to maintain a constant total amount of Br^⁻^ to ensure that the sizes of the QDs were not affected by the amount of Mn doping. ICP-MS analyses showed that the concentration of Zn^2+^ was extremely low, which indicated that Zn^2+^ was not effectively doped into the QDs [19].

For single QD measurements, the hexane solution of perovskite QDs was mixed with 1 wt.% polystyrene solution. The mixture was then spin-coated onto clean glass coverslips at 3000 rpm for one minute to form a polystyrene film to protect the perovskite QDs. The size of the perovskite QDs was measured from the TEM image using a JEM-2100 microscope (JEOL, Tokyo, Japan). The absorption, excitation, and PL emission spectra of the perovskite QDs in hexane were measured using a PerkinElmer Lambda 950 UV–VISNIR spectrometer (PerkinElmer, Hopkinton, MA, USA) and a Cary Eclipse Fluorescence Spectrophotometer (Agilent, Santa Clara, CA, USA), respectively. X-ray diffraction (XRD) patterns of the perovskite QDs were obtained using a D2 PHASER diffractometer (Bruker, Billerica, MA, USA). The elemental concentrations of Mn-doped perovskite QDs were determined using a NexION350 series inductively coupled plasma mass spectrometer (ICP-MS) (PerkinElmer, MA, USA).

A confocal fluorescence microscopy system was home-built to collect the PL photons emitted from single QDs [27,28]. A pulsed laser emitting at 439 nm (EXW-12, NKT) with a pulse duration of 50 to 100 ps and a repetition rate of 5 MHz was used for excitation. The system incorporated an Olympus oil immersion objective with a high magnification of 100× and a numerical aperture (NA) of 1.3, which not only facilitated the precise focusing of the laser beam onto the QD samples but also efficiently collects the emitted PL photons. The collected PL photons were initially filtered through a dichroic mirror (Semrock, IDEX, Northbrook, IL, USA) and a long-pass filter (Semrock, IDEX, IL, USA) to eliminate any background noise that might otherwise interfere with the analysis. Subsequently, a spatial filtering step using a 100 μm pinhole was employed to reject out-of-focus photons, thereby ensuring that only the desired PL photons were processed. A 50/50 beam-splitter cube was then used to divide the filtered PL photons into two equal beams. Each beam was then detected by a highly sensitive single-photon avalanche diode detector (SPCM-AQR-15, PerkinElmer, MA, USA), which is capable of rapidly responding to individual photons. The detected signals were accurately recorded by a time-tagged, time-resolved (TTTR), time-correlated single-photon counting (TCSPC) data acquisition card (HydraHarp 400, PicoQuant, Berlin, Germany), which provides a temporal resolution of 1 ps. Of note, all measurements were conducted at room temperature in order to ensure reproducibility and comparability with other experiments. The described setup and procedures adhere to standard practices in fluorescence microscopy and single-photon detection, thus ensuring the reliability and accuracy of the data obtained for the investigation of the PL properties of perovskite QDs.

## 3. Results and Discussion

**Figure 1:**
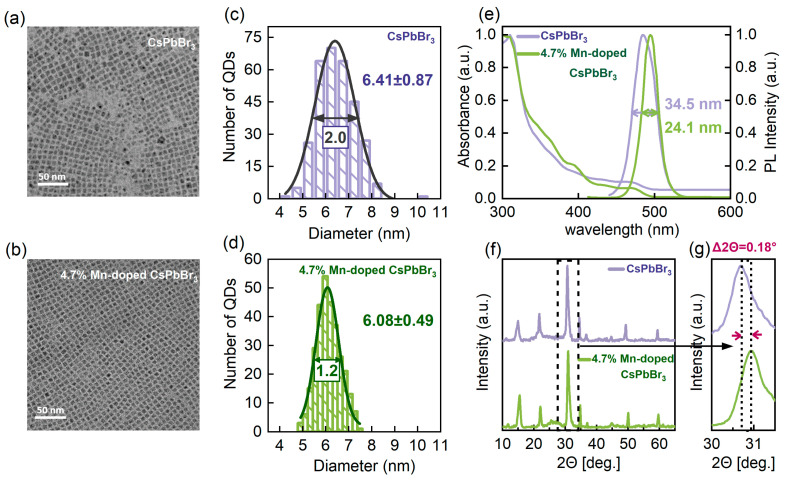
Morphological, spectral, and structural characterization of undoped and 4.7% Mn-doped CsPbBr_3_ perovskite quantum dots (QDs). (**a**,**b**) Transmission electron microscopy (TEM) images of undoped and Mn-doped CsPbBr_3_ perovskite QDs. (**c**,**d**) Histograms depicting the distribution of edge lengths for undoped and Mn-doped perovskite QDs. (**e**) Absorption and PL emission spectra of undoped and Mn-doped perovskite QDs, demonstrating that Mn-doped perovskite QDs exhibit a narrower linewidth. (**f**) X-ray diffraction (XRD) patterns of perovskite QDs with different Mn doping concentrations, demonstrating the preservation of the cubic phase upon Mn doping. (**g**) A magnified view shows a subtle but monotonic shift in the XRD peak toward higher 2θ angles, indicating a progressive contraction of the lattice with Mn doping.

The synthesis of the Mn-doped CsPbBr_3_ perovskite QDs was successfully accomplished using MnBr_2_ as a dopant. The doping concentrations of the QDs were 4.7%, 20.4%, and 42.9%, with the undoped QDs serving as a control. The Mn^2+^ concentration was accurately determined by inductively coupled plasma mass spectrometry (ICP-MS) analysis, as shown in Table S2. Taking the undoped and the 4.7% Mn-doped CsPbBr_3_ perovskite QDs as examples, the solution PL quantum yield (QY) of the undoped QDs is about 80%, and the PLQY of the 4.7% doped QDs is about 70%. The transmission electron microscopy (TEM) images presented in Figure 1a,b illustrate the distinct morphological discrepancies between the undoped and the 4.7% Mn-doped perovskite QDs. Following the incorporation of 4.7% Mn doping, notable improvements in both the size distribution and morphology of the perovskite QDs were observed. In particular, these QDs exhibit a strikingly uniform size distribution, with a significant reduction in the full width at half maximum (FWHM) of the size distribution from 2 nm to 1.2 nm (Figure 1c,d), which highlights the high level of control over the size uniformity obtained by Mn doping. Furthermore, the original cubic morphology becomes more distinct and well-defined, indicating an improvement in crystallinity. The size narrowing can be attributed to the effective modulation of QD surface defects by Mn doping [26,29]. Mn doping has the effect of reducing surface defects by increasing the defect formation energy while avoiding the introduction of deep traps into the band gap. The reduction in surface defects not only improves the short-range ordering of the lattice but also brings the local structural ordering closer to the ideal homogeneous state. At the same time, this optimization is reflected in the emission spectrum of the perovskite QDs, where the FWHM of the emission spectrum is significantly narrowed from 34.5 nm to 24.1 nm (Figure 1e), representing a 30.3% reduction as compared to the original width. This indicates a more homogeneous energy distribution and the improved optical properties of the doped perovskite QDs, which can be attributed to the profound effect of Mn doping in regulating and reducing surface defects on the QD surfaces. Conventionally, during the growth process of perovskite QDs, the presence of unpaired chemical bonds leads to the formation of surface defects with high free energy, acting as non-radiative recombination centers that deteriorate the optical performance. However, the introduction of Mn^2+^ as a dopant effectively mitigates this issue by increasing the defect formation energy barrier, thereby suppressing the formation of such defects and avoiding the introduction of deep traps within the band gap. This mechanism not only enhances the short-range order of the crystal lattice but also brings the local structural order closer to an ideal state of uniformity, as evidenced by the improved optical properties [26,29]. Furthermore, the substitution of Pb^2+^, which possesses a larger ionic radius, by the equivalent but smaller Mn^2+^, lead to a contraction of the lattice. This lattice contraction not only contributes to the observed reduction in the average edge length of the perovskite QDs from 6.41 nm to 6.08 nm (Figure 1c,d), but also affects the electronic structure. Specifically, the enhancement of the coordination field around Mn^2+^ due to lattice contraction results in a narrowing of the band gap between the ^4^T_1_ and ^6^A_1_ energy levels of Mn^2+^, leading to a slight redshift in the position of the PL excitation and emission peaks (Figure 1e and Figure S1) [30]. This effect, coupled with the reduced surface defects, underscores the beneficial role of Mn doping in enhancing the optical properties of the perovskite QDs. Additionally, the monotonic shift of the peaks in the X-ray diffraction (XRD) patterns by 0.18° toward higher angles (Figure 1f,g) provides further evidence of the lattice contraction induced by Mn doping. The XRD plots of perovskite QDs with different doping concentrations, along with comparisons to the standard phase, are provided in Supplementary Materials Figure S2. The observed improvements in energy distribution, optical properties, and structural order of the doped perovskite QDs provide compelling evidence that Mn doping effectively regulates and reduces surface defects, leading to enhanced performance. These findings not only validate the successful doping of the perovskite QDs but also highlight the potential of Mn^2+^ as a powerful dopant for improving the properties of semiconductor nanomaterials.

As the doping concentration is increased from 4.7% to 20.4%, a slight blue shift in the absorption edge can be observed, accompanied by a corresponding shift in the PL peak from 496.6 nm to 486.6 nm. When the doping concentration exceeds 42.9%, the size distribution and morphology of the perovskite QDs deteriorate, resulting in a reverse redshift of the characteristic spectra (Figure S3g,h and Table S3). Furthermore, it was observed that the Mn-doped perovskite QDs did not exhibit Mn-related PL in the visible spectral region at room temperature due to the low energy of the excitonic transition [30,31,32]. 

**Figure 2:**
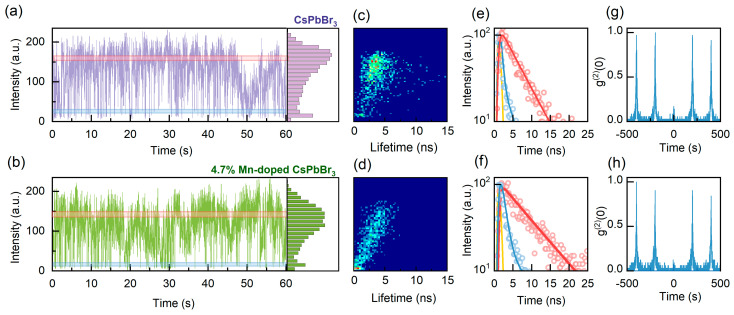
(**a**,**b**) Typical PL intensity time trajectories for single undoped and 4.7% Mn-doped CsPbBr_3_ perovskite QDs. The corresponding PL intensity histograms are presented in the right panels. (**c**,**d**) Corresponding fluorescence lifetime–intensity distribution (FLID) maps. The color change from blue to red indicates an increase in the probability of occurrence of a given state in the intensity–lifetime space. (**e**,**f**) Corresponding PL decay trajectories obtained from the bright- and dim-state PL regions marked in respective colors on the PL intensity trajectories of (**a**,**b**). The solid red and blue lines represent mono-exponential fits, and the solid yellow lines in the figures represent the instrument response function (IRF) of the system. (**g**,**h**) Corresponding second-order correlation function ($g^{\left(2\right)}$) curves of the bright-state PL regions for single undoped and 4.7% Mn-doped CsPbBr_3_ perovskite QDs.

In order to investigate the impact of doping on CsPbBr_3_ perovskite QDs, we conducted single-dot PL spectroscopy on undoped and 4.7% Mn-doped CsPbBr_3_ perovskite QDs under identical excitation conditions. The excitation condition 〈*N*〉 represents the average number of photons absorbed per QD per pulse. 〈*N*〉 = σ·*j*_exc_, where *j*_exc_ is the per-pulse photon fluence, and σ is the QD absorption cross section [4,33]. The estimation of 〈*N*〉 and the calculation of the absorption cross section σ can be found in the Supplementary Materials. The average values of σ for the undoped CsPbBr_3_ perovskite QDs and the 4.7% Mn-doped CsPbBr_3_ perovskite QDs are 3.52 × 10^−14^ cm^2^ and 2.97 × 10^−14^ cm^2^, respectively (Figure S4), which is consistent with the findings of previous research [4]. Figure 2a,b illustrate the typical PL intensity trajectories for single undoped and 4.7% Mn-doped perovskite QDs under low excitation (〈*N*〉 = 0.2). The corresponding PL intensity histograms are shown in the right panels. From the figures, it can be observed that the PL intensity trajectory of the single 4.7% Mn-doped QD remains essentially unchanged as compared to that of the undoped QD. However, as the doping concentration increases, the PL trajectories of the perovskite QDs deteriorate, exhibiting a decrease in intensity and an increase in blinking (Figure S5). By applying a single exponential fit to each 10 ms time bin of the PL trajectories, fluorescence lifetime-intensity distribution (FLID) maps can be derived, which provide valuable insights into the PL properties of the QDs [34,35]. Prior to and following doping, the PL intensity of single CsPbBr_3_ perovskite QDs exhibits a linear correlation with their lifetimes, indicating that PL blinking in both the undoped and Mn-doped QDs is primarily dominated by band-edge carrier (BC) blinking (Figure 2c,d). This implies that the moderate amount of Mn doping did not alter the blinking mechanism of the perovskite QDs.

The corresponding PL decay trajectories, extracted from the bright- and dim-state PL regions, are presented in Figure 2e,f, respectively. The decay curves can be fitted by mono-exponential functions to obtain the bright- and dim-state lifetimes. The radiative lifetime scaling can be calculated based on the bright- and dim-state lifetimes and the corresponding PL intensities of the bright- and dim-state. The radiative lifetime scaling for both the undoped and 4.7% Mn-doped CsPbBr_3_ perovskite QDs is approximately 1.0 (details of the scaling calculations can be found in the Supplementary Materials), which further confirms that both undoped and Mn-doped perovskite QDs adhere to the BC blinking mechanism [36,37]. This BC blinking behavior indicates that the blinking of the undoped and Mn-doped perovskite QDs is primarily attributed to the surface-shallow trap states. Given the analogous energy level structure of the Mn and CsPbBr_3_ perovskite QDs, doping Mn²⁺ does not introduce deep trap states and thus does not alter the carrier trapping and de-trapping rates. The corresponding second-order correlation function ($g^{\left(2\right)}$) curves depicted in Figure 2g,h show very low values of $g^{\left(2\right)}(0)$, well below 0.5. This observation suggests that the investigated perovskite QDs are single QDs [38] and also indicates that Mn doping does not appear to alter the photon statistical properties of the QDs. 

**Figure 3:**
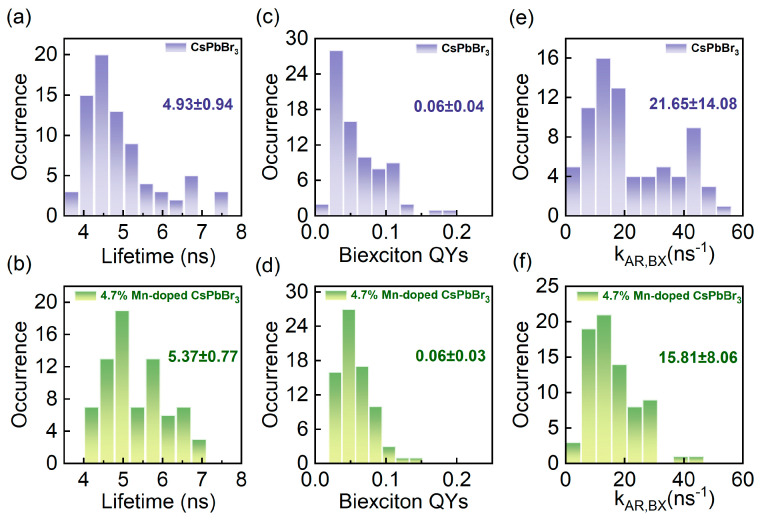
(**a**,**b**) Histograms of single-exciton lifetimes for single undoped and 4.7% Mn-doped CsPbBr_3_ perovskite QDs. (**c**,**d**) Histograms of biexciton quantum yields (QYs) for single undoped and Mn-doped CsPbBr_3_ perovskite QDs. (**e**,**f**) Histograms of biexciton Auger recombination rates ($k_{AR,BX}$) for single undoped and Mn-doped CsPbBr_3_ perovskite QDs.

In order to gain further insight into the impact of Mn doping on CsPbBr_3_ perovskite QDs, a statistical analysis was conducted on the single-exciton lifetimes of both single undoped and Mn-doped CsPbBr_3_ perovskite QDs. The single-exciton lifetime can be determined by fitting the bright state of the PL intensity trajectory. The histograms of single-exciton lifetimes are presented in Figure 3a,b, and the results were obtained from approximately 100 QDs in each case. The average lifetime value of 4.7% Mn-doped CsPbBr_3_ perovskite QDs is 5.37 ns, which is larger than the average lifetime value of undoped CsPbBr_3_ perovskite QDs (4.93 ns). This increase in the lifetime value indicates that the doping of Mn²⁺ favors the suppression of defect recombination [29]. 

Furthermore, we conducted a detailed investigation into the impact of doping on the biexciton quantum yields (QYs) and biexciton Auger recombination rates ($k_{AR,BX}$) in perovskite QDs. In the case of weak excitation conditions with 〈*N*〉 = 0.2, the biexciton QYs can be calculated using the Equation (1) [39,40]:

(1) $$QY_{BX}=g^{\left(2\right)}\left(0\right)\times QY_{X}$$

where $QY_{BX}$ and $QY_{X}$ are the biexciton QY and the single-exciton QY, respectively. It is assumed that the single-exciton QY of the on-state PL intensity trajectory is unity for single QDs [39]. According to the values of $g^{\left(2\right)}(0)$, the biexciton QYs of single undoped and Mn-doped CsPbBr_3_ perovskite QDs can be obtained by Equation (1). The histograms of the biexciton QYs in the two cases are presented in Figure 3c,d, respectively, and the average values are calculated to be 0.06 and 0.06, respectively. This suggests that Mn doping has a minimal impact on the biexciton QYs of perovskite QDs. 

When the excitation condition 〈*N*〉 ≪ 1, the relationship of $g^{\left(2\right)}(0)$ to the biexciton and single-exciton lifetimes of a single perovskite QD can be expressed by Equation (2) [16,40,41]:

(2) $$g^{\left(2\right)}\left(0\right)=4\frac{\tau_{BX}}{\tau_{X}}$$

where $\tau_{BX}$ and $\tau_{X}$ are the biexciton and single-exciton lifetimes, respectively. The biexciton QY can be expressed by Equation (3) [40]:

(3) $$QY_{BX}=\frac{k_{r,BX}}{k_{r,BX}+k_{AR,BX}}=\frac{k_{r,BX}}{1∕\tau_{BX}}$$

where $k_{r,BX}$ and $k_{AR,BX}$ represent the biexciton radiative rate and the biexciton Auger recombination rate, respectively. According to Equations (2) and (3), $k_{AR,BX}$ can be obtained. Histograms of $k_{AR,BX}$ for single undoped and Mn-doped CsPbBr_3_ perovskite QDs are shown in Figure 3e,f. The $k_{AR,BX}$ values for undoped perovskite QDs exhibit a relatively wide distribution, reflecting the non-uniform size distribution of the undoped QDs (Figure 3e). The mean value of $k_{AR,BX}$ is 21.65 ns^−1^. In contrast, the distribution of $k_{AR,BX}$ of the Mn-doped perovskite QDs is more concentrated (Figure 3f). This is primarily attributed to the enhancement of their size uniformity and the significant reduction in size dispersion achieved by Mn doping. The average value of $k_{AR,BX}$ is 15.81 ns^−1^ for the Mn-doped QDs, which is smaller than that of the undoped QDs. This evidence demonstrates that the incorporation of Mn effectively mitigates the discreteness of the $k_{AR,BX}$. The reduction in the $k_{AR,BX}$ achieved by minimizing non-radiative recombination processes leads to an increase in the radiative recombination fraction. At the same time, the reduced $k_{AR,BX}$ indicates that the quantum confinement on the carriers is reduced, creating favorable conditions for the overall enhancement of the LED device performance. A summary table of photophysical parameters for the Mn-doped QDs obtained in this work is presented in Table S3, and results from the other literature used for comparison are also included in Table S3. In addition, the 4.7% Mn-doped QDs met our expectations, so we did not attempt to prepare QDs with small changes in the Mn concentration.

**Figure 4:**
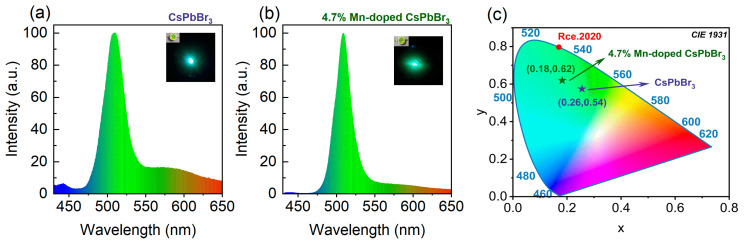
(**a**,**b**) Light-emitting diode (LED) emission spectra of the undoped and 4.7% Mn-doped CsPbBr_3_ perovskite QDs. Insets: Optical images corresponding to the LED. (**c**) Chromaticity coordinates.

One of the key challenges in the advancement of QD LED (QLED) technology is the relatively wide emission spectra, which affects the color purity of the emitted light from LEDs [42,43]. To further validate the feasibility of Mn-doped CsPbBr_3_ perovskite QDs for LED applications, we employed undoped and 4.7% Mn-doped CsPbBr_3_ perovskite QDs as down-conversion phosphors. The CsPbBr_3_ perovskite QDs were thoroughly mixed with a 1wt% polystyrene (PS)-toluene solution to improve the stability. The resulting mixture was then applied to a 395 nm UV chip and allowed to dry in air for one hour, resulting in the fabrication of the QLED device. As illustrated in Figure 4a,b, at a driving voltage of 3 V, the FWHM of the light emission of the QLED based on Mn-doped CsPbBr_3_ perovskite QDs is 24.3 nm, which is smaller than that of the undoped QDs (31.5 nm). The green light purity of the two QLEDs is expressed in CIE coordinates as (0.26, 0.54) for the undoped perovskite QDs and (0.18, 0.62) for the 4.7% Mn-doped perovskite QDs, as shown in Figure 4c. Notably, the QLED based on the Mn-doped QDs exhibit color coordinates that are closer to the ideal green LED coordinates in the 1931 color space (0.170, 0.797) [44] and exhibit a higher degree of color purity, which highlights the great potential of doped perovskite QDs for LED fabrication.

## 4. Conclusions

Mn-doped CsPbBr_3_ perovskite QDs were successfully prepared by introducing an appropriate amount of Mn²⁺ into the QDs. The study demonstrates that Mn doping significantly increases the defect formation energy, which promotes the formation of nearly uniform local structural order in the perovskite QDs, leading to improved size uniformity and monodispersity. The FWHM of the size distribution of the perovskite QDs was reduced from 2 nm to 1.2 nm, with a 30.3% reduction in the PL emission linewidth as compared to the undoped QDs, indicating a more homogeneous energy distribution and improved optical properties. The study reveals that the optimal doping effect is achieved when the Mn doping concentration of perovskite QDs is set at 4.7% among four different doping concentrations. This doping process does not result in the introduction of deep trap states within the band gap and instead improves the size uniformity. Furthermore, the improvement in PL properties is evidenced by the narrowing of the spectral linewidths, the increase in PL lifetime, and the reduction in the Auger recombination rate. Additionally, a 23% reduction in linewidth was observed for the 4.7% doped QLEDs, with a CIE color coordinate of (0.18, 0.62) that is closer to the ideal coordinates for green LEDs, further demonstrating their potential for LED applications. This work offers an effective strategy for enhancing the color purity of perovskite QD-based LEDs.

## Data Availability

Data underlying the results are contained within the article or the Supplementary Materials.

## Supplementary Materials

The following supporting information can be downloaded at https://www.mdpi.com/article/10.3390/nano14151284/s1: Estimation of 〈*N*〉 and calculation of the absorption cross section; BC blinking determined by radiative lifetime scaling; Figure S1: excitation spectra of PL for undoped and 4.7% Mn-doped perovskite CsPbBr3 QDs; Figure S2: X-ray diffraction (XRD) patterns of perovskite QDs with different Mn doping concentrations; Figure S3: morphological and structural characterizations of undoped and Mn-doped CsPbBr3 perovskite QDs with various doping concentrations; Figure S4: histograms of the absorption cross sections (σ) of undoped and 4.7% Mn-doped CsPbBr3 perovskite QDs; Figure S5: typical PL intensity time trajectory for single CsPbBr3 perovskite QDs with different doping concentrations; Table S1: mass and molar ratios of PbBr2, MnBr2 and ZnBr2 at different doping concentrations; Table S2: quantitative elemental scanning ICP-OES analysis of Mn-doped CsPbBr3 QDs under different doping concentrations; and Table S3: a summary table of photophysical parameters for the Mn-doped QDs obtained in this work and results from other literature used for comparison. References [45,46,47] are cited in the Supplementary Materials.

## References

[1] Tang Y., Tang S., Luo M., Guo Y., Zheng Y., Lou Y., Zhao Y. (2021). All-inorganic lead-free metal halide perovskite quantum dots: Progress and prospects. Chem. Commun..

[2] Wu X., Ji H., Yan X., Zhong H. (2022). Industry outlook of perovskite quantum dots for display applications. Nat. Nanotechnol..

[3] Li B., Zhang G., Gao Y., Chen X., Chen R., Qin C., Hu J., Wu R., Xiao L., Jia S. (2024). Single quantum dot spectroscopy for exciton dynamics. Nano Res..

[4] Li B., Huang H., Zhang G., Yang C., Guo W., Chen R., Qin C., Gao Y., Biju V.P., Rogach A.L. (2018). Excitons and biexciton dynamics in single CsPbBr_3_ perovskite quantum dots. J. Phys. Chem. Lett..

[5] Li Y., Du K., Zhang M., Gao X., Lu Y., Yao S., Li C., Feng J., Zhang H. (2021). Tunable ultra-uniform Cs_4_PbBr_6_ perovskites with efficient photoluminescence and excellent stability for high-performance white light-emitting diodes. J. Mater. Chem..

[6] He X., Li T., Liang Z., Liu R., Ran X., Wang X., Guo L., Pan C. (2024). Enhanced cyan photoluminescence and stability of CsPbBr_3_ quantum dots via surface engineering for white light-emitting diodes. Adv. Opt. Mater..

[7] Liu Y., Yuan S., Zheng H., Wu M., Zhang S., Lan J., Li W., Fan J. (2023). Structurally dimensional engineering in perovskite photovoltaics. Adv. Energy Mater..

[8] Chen L.J., Dai J.H., Lin J.D., Mo T.S., Lin H.P., Yeh H.C., Chuang Y.C., Jiang S.A., Lee C.R. (2018). Wavelength-tunable and highly stable perovskite-quantum-dot-doped lasers with liquid crystal lasing cavities. ACS Appl. Mater. Interfaces.

[9] Nguyen T., Tan L.Z., Baranov D. (2023). Tuning perovskite nanocrystal superlattices for superradiance in the presence of disorder. J. Chem. Phys..

[10] Lin L., Liu Y., Wu W., Huang L., Zhu X., Xie Y., Liu H., Zheng B., Liang J., Sun X. (2023). Self-powered perovskite photodetector arrays with asymmetric contacts for imaging applications. Adv. Electron. Mater..

[11] Pan X., Ding L. (2022). Application of metal halide perovskite photodetectors. J. Semicond..

[12] Ma J., Zhang J., Horder J., Sukhorukov A.A., Toth M., Neshev D.N., Aharonovich I. (2024). Engineering quantum light sources with flat optics. Adv. Mater..

[13] Kulkarni S.A., Yantara N., Tan K.S., Mathews N., Mhaisalkar S.G. (2020). Perovskite nanostructures: Leveraging quantum effects to challenge optoelectronic limits. Mater. Today.

[14] Lu X., Hou X., Tang H., Yi X., Wang J. (2022). A high-quality CdSe/CdS/ZnS quantum-dot-based FRET aptasensor for the simultaneous detection of two different Alzheimer’s disease core biomarkers. Nanomaterials.

[15] Chen M., Shen G., Guyot-Sionnest P. (2020). Size distribution effects on mobility and intraband gap of HgSe quantum dots. J. Phys. Chem. C.

[16] Yang C., Zhang G., Gao Y., Li B., Han X., Li J., Zhang M., Chen Z., Wei Y., Chen R. (2024). Size-dependent photoluminescence blinking mechanisms and volume scaling of biexciton Auger recombination in single CsPbI_3_ perovskite quantum dots. J. Chem. Phys..

[17] Bai X., Li H., Peng Y., Zhang G., Yang C., Guo W., Han X., Li J., Chen R., Qin C. (2022). Role of aspect ratio in the photoluminescence of single CdSe/CdS dot-in-rods. J. Phys. Chem. C.

[18] Korepanov O., Kozodaev D., Aleksandrova O., Bugrov A., Firsov D., Kirilenko D., Mazing D., Moshnikov V., Shomakhov Z. (2023). Temperature-and size-dependent photoluminescence of CuInS_2_ quantum dots. Nanomaterials.

[19] Dong Y., Qiao T., Kim D., Parobek D., Rossi D., Son D.H. (2018). Precise control of quantum confinement in cesium lead halide perovskite quantum dots via thermodynamic equilibrium. Nano Lett..

[20] Larson H., Cossairt B.M. (2023). Indium-poly (carboxylic acid) ligand interactions modify InP quantum dot nucleation and growth. Chem. Mater..

[21] Paik T., Greybush N.J., Najmr S., Woo H.Y., Hong S.V., Kim S.H., Lee J.D., Kagan C.R., Murray C.B. (2024). Shape-controlled synthesis and self-assembly of highly uniform upconverting calcium fluoride nanocrystals. Inorg. Chem. Front..

[22] Chen J., Zhang L., Li S., Jiang F., Jiang P., Liu Y. (2021). Cu-deficient cuinse quantum dots for “turn-on” detection of adenosine triphosphate in living cells. ACS Appl. Nano Mater..

[23] Akkerman Q.A., Park S., Radicchi E., Nunzi F., Mosconi E., De Angelis F., Brescia R., Rastogi P., Prato M., Manna L. (2017). Nearly monodisperse insulator Cs_4_PbX_6_ (X = Cl, Br, I) nanocrystals, their mixed halide compositions, and their transformation into CsPbX_3_ nanocrystals. Nano Lett..

[24] Kirakosyan A., Kim Y., Sihn M.R., Jeon M.G., Jeong J.R., Choi J. (2020). Solubility-controlled room-temperature synthesis of cesium lead halide perovskite nanocrystals. ChemNanoMat.

[25] Manoli A., Papagiorgis P., Sergides M., Bernasconi C., Athanasiou M., Pozov S., Choulis S.A., Bodnarchuk M.I., Kovalenko M.V., Othonos A. (2021). Surface functionalization of CsPbBr_3_ nanocrystals for photonic applications. ACS Appl. Nano Mater..

[26] Yong Z., Guo S., Ma J., Zhang J., Li Z., Chen Y., Zhang B., Zhou Y., Shu J., Gu J. (2018). Doping-enhanced short-range order of perovskite nanocrystals for near-unity violet luminescence quantum yield. J. Am. Chem. Soc..

[27] Li J., Wang D., Zhang G., Yang C., Guo W., Han X., Bai X., Chen R., Qin C., Hu J. (2022). The role of surface charges in the blinking mechanisms and quantum-confined stark effect of single colloidal quantum dots. Nano Res..

[28] Li B., Gao Y., Wu R., Miao X., Zhang G. (2023). Charge and energy transfer dynamics in single colloidal quantum dots/monolayer MoS_2_ heterostructures. Phys. Chem. Chem. Phys..

[29] Bi C., Wang S., Li Q., Kershaw S.V., Tian J., Rogach A.L. (2019). Thermally stable copper(II)-doped cesium lead halide perovskite quantum dots with strong blue emission. J. Phys. Chem. Lett..

[30] Mir W.J., Mahor Y., Lohar A., Jagadeeswararao M., Das S., Mahamuni S., Nag A. (2018). Postsynthesis doping of Mn and Yb into CsPbX_3_ (X = Cl, Br, or I) perovskite nanocrystals for downconversion emission. Chem. Mater..

[31] Liu W., Lin Q., Li H., Wu K., Robel I., Pietryga J.M., Klimov V.I. (2016). Mn^2+^-doped lead halide perovskite nanocrystals with dual-color emission controlled by halide content. J. Am. Chem. Soc..

[32] Yun R., Yang H., Sun W., Zhang L., Liu X., Zhang X., Li X. (2023). Recent advances on Mn^2+^-doping in diverse metal halide perovskites. Laser Photonics Rev..

[33] Hu F., Zhang H., Sun C., Yin C., Lv B., Zhang C., Yu W.W., Wang X., Zhang Y., Xiao M. (2015). Superior optical properties of perovskite nanocrystals as single photon emitters. ACS Nano.

[34] Han X., Zhang G., Li B., Yang C., Guo W., Bai X., Huang P., Chen R., Qin C., Hu J. (2020). Blinking mechanisms and intrinsic quantum-confined stark effect in single methylammonium lead bromide perovskite quantum dots. Small.

[35] Yang C., Li Y., Hou X., Zhang M., Zhang G., Li B., Guo W., Han X., Bai X., Li J. (2023). Conversion of photoluminescence blinking types in single colloidal quantum dots. Small.

[36] Podshivaylov E.A., Kniazeva M.A., Tarasevich A.O., Eremchev I.Y., Naumov A.V., Frantsuzov P.A. (2023). A quantitative model of multi-scale single quantum dot blinking. J. Mater. Chem..

[37] Yuan G., Gómez D.E., Kirkwood N., Boldt K., Mulvaney P. (2018). Two mechanisms determine quantum dot blinking. ACS Nano.

[38] Li B., Zhang G., Yang C., Li Z., Chen R., Qin C., Gao Y., Huang H., Xiao L., Jia S. (2018). Fast recognition of single quantum dots from high multi-exciton emission and clustering effects. Opt. Express.

[39] Nair G., Zhao J., Bawendi M.G. (2011). Biexciton quantum yield of single semiconductor nanocrystals from photon statistics. Nano Lett..

[40] Guo W., Tang J., Zhang G., Li B., Yang C., Chen R., Qin C., Hu J., Zhong H., Xiao L. (2021). Photoluminescence blinking and biexciton Auger recombination in single colloidal quantum dots with sharp and smooth core/shell interfaces. J. Phys. Chem. Lett..

[41] Park Y., Bae W.K., Padilha L.A., Pietryga J.M., Klimov V.I. (2014). Effect of the core/shell interface on Auger recombination evaluated by single-quantum-dot spectroscopy. Nano Lett..

[42] Zhuang X., Liang B., Jiang C., Wang S., Bi H., Wang Y. (2024). Narrow band organic emitter for pure green solution-processed electroluminescent devices with CIE coordinate y of 0.77. Adv. Opt. Mater..

[43] Li S., Pan J., Yu Y., Zhao F., Wang Y., Liao L. (2023). Advances in solution-processed blue quantum dot light-emitting diodes. Nanomaterials.

[44] Kumar S., Jagielski J., Kallikounis N., Kim Y., Wolf C., Jenny F., Tian T., Hofer C.J., Chiu Y., Stark W.J. (2017). Ultrapure green light-emitting diodes using two-dimensional formamidinium perovskites: Achieving recommendation 2020 color coordinates. Nano Lett..

[45] Duan W., Hu L., Zhao W., Zhang X. (2022). Rare-earth ion-doped perovskite quantum dots: Synthesis and optoelectronic properties. J. Mater. Sci. Mater. Electron..

[46] Biswas A., Bakthavatsalam R., Kundu J. (2017). Efficient exciton to dopant energy transfer in Mn^2+^-doped (C_4_H_9_NH_3_)_2_PbBr_4_ two-dimensional (2D) layered perovskites. Chem. Mater..

[47] Zeng F., Tan Y., Hu W., Tang X., Zhang X., Yin H. (2022). A facile strategy to synthesize high colour purity blue luminescence aluminium-doped CsPbBr3 perovskite quantum dots. J. Lumin..

