# Supplementary material for: Size Uniformity of CsPbBr3 Perovskite Quantum Dots via Manganese-Doping
Source: Nanomaterials (Basel). 2024 Jul 30;14(15):1284. doi: 10.3390/nano14151284 (PMC11313879; doi:10.3390/nano14151284)
Supplement: Supplementary file 1 [file nanomaterials-14-01284-s001.zip › nanomaterials-3116237-supplementary.pdf]

nanomaterials

Supplementary Materials

Size uniformity of $CsPbBr_3$ perovskite quantum dots via manganese-doping

Mi Zhang [1], Xue Han [1], Changgang Yang [1], Guofeng Zhang [1,*], Wenli Guo [1], Jialu Li [1], Zhihao Chen [1], Bin Li [1], Ruiyun Chen [1], Chengbing Qin [1], Jianyong Hu [1], Zhichun Yang [1], Ganying Zeng [1], Liantuan Xiao [1,2,*], and Suotang Jia [1]

[1] State Key Laboratory of Quantum Optics and Quantum Optics Devices, Institute of Laser Spectroscopy, Collaborative Innovation Center of Extreme Optics, Shanxi University, Taiyuan, 030006, China
[2] College of Physics, Taiyuan University of Technology, Taiyuan, Shanxi 030006, China
* Correspondence: guofeng.zhang@sxu.edu.cn; xlt@sxu.edu.cn

Estimation of $\langle N \rangle$ and calculation of the absorption cross section σ :

We can estimate the average number of absorbed photons per quantum dots (QDs) per pulse $\langle N \rangle$ for a single perovskite QD by using the following equation:

$$\langle N \rangle = \frac{I_x}{F \cdot \xi \cdot \eta_x} \quad ,$$

where I_x represents the PL count rate of the exciton, F is the repetition rate of the pump pulse (5 HMz), η_x is the PLQY of the exciton, and ξ represents the detection efficiency of the system.

Since the perovskite QDs are embedded in polystyrene, the environmental effects can be mitigated. Assuming a η_x of 1 at the highest intensity level, the $\langle N \rangle$ can be accurately estimated based on the PL intensity trace, particularly at lower excitation powers. Subsequently, the absorption cross-section σ can be calculated using this estimation,

$$\langle N \rangle = \sigma \times j_{exc} \quad ,$$

where j_{exc} is the excitation photon flux (photons/cm^2), which can be calculated by the following equation:

$$j_{exc} = \frac{I_{excitation}}{(hv \times F)},$$

where $I_{excitation}$ is the power density of the laser excitation (W/cm^2), neglecting the effect of higher order excitons, and the photoluminescence intensity $I_{excitation}$ is proportional to $\langle N \rangle$:

$$I_{excitation} \propto 1 - e^{-\langle N \rangle}.$$

The equation can finally be expressed as:

$$\langle N \rangle = \sigma \times \frac{I_{excitation}}{(hv \times F)}.$$

For undoped and Mn-doped $CsPbBr_3$ perovskite QDs, the average value of σ can be calculated to be 3.52×10^{-14} cm^2 and 2.97×10^{-14} cm^2, respectively.

Calculations of radiative lifetime scaling:

The radiative lifetime scaling for the two PL intensity regions of single perovskite QDs is calculated as follows:

$$\tau_1 : \tau_2 = \frac{\tau_1}{I_1} : \frac{\tau_2}{I_2} = \frac{3.7114}{168 - 3(background)} : \frac{0.6666}{31 - 3(background)} \approx 1.058$$

For a single Mn-doped $CsPbBr_3$ perovskite QD, the radiative lifetime scaling for the two PL intensity regions is also calculated as follows:

$$\tau_1 : \tau_2 = \frac{\tau_1}{I_1} : \frac{\tau_2}{I_2} = \frac{5.4088}{163 - 2(background)} : \frac{1.1804}{28 - 2(background)} \approx 0.740$$

Calculations of doping concentration:

The doping concentrations can be determined based on ICP test results, and are calculated as follows: doping concentration = molar concentration of Mn / (molar concentration of Pb + molar concentration of Mn). The doping concentrations of the three Mn-doped $CsPbBr_3$ QD samples were calculated to be 4.7%, 20.4%, and 42.9%, respectively.

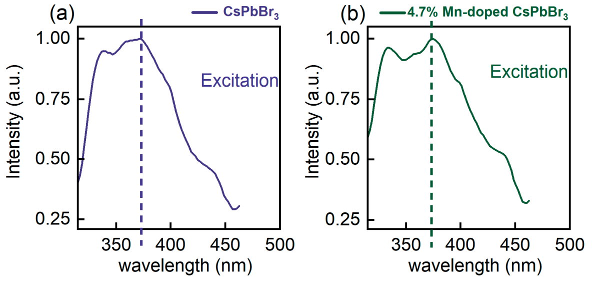

Figure S1: (a, b) Excitation spectra of PL for undoped and 4.7% Mn-doped perovskite $CsPbBr_3$ QDs.

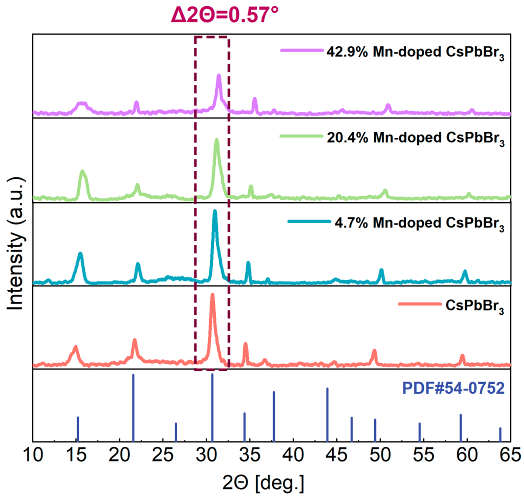

Figure S2: X-ray diffraction (XRD) patterns of perovskite QDs with different Mn doping concentrations.

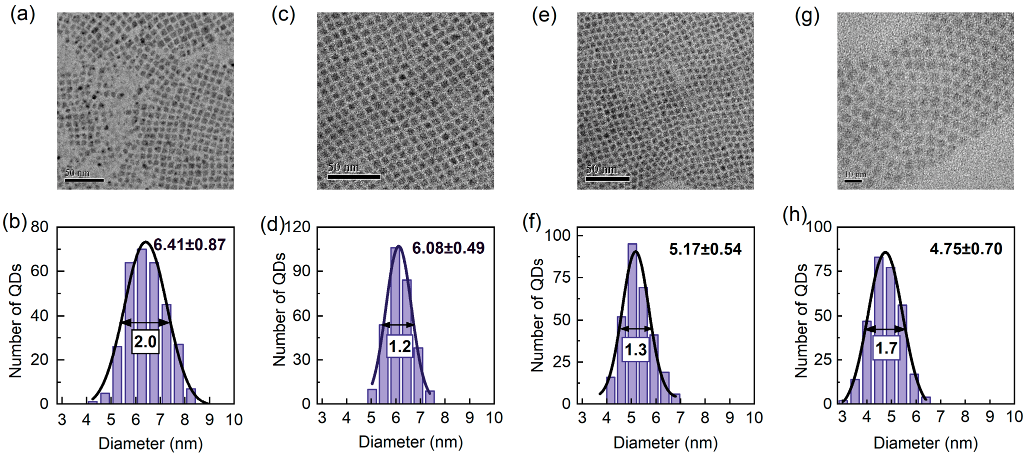

Figure S3: Morphological and structural characterizations of undoped and Mn-doped $CsPbBr_3$ perovskite QDs with various doping concentrations. Transmission electron microscopy (TEM) images, and histograms of edge length distribution with the corresponding Gaussian fitting curve for undoped $CsPbBr_3$ perovskite QDs (a-b), 4.7% Mn-doped $CsPbBr_3$ perovskite QDs (c-d), 20.4% Mn-doped $CsPbBr_3$ perovskite QDs (e-f), and 42.9% Mn-doped $CsPbBr_3$ perovskite QDs (g-h). The doping concentration of 4.7% results in the best uniformity of the QD sizes. Excessive addition of Mn will have an adverse effect on the size distribution and stability of the QDs.

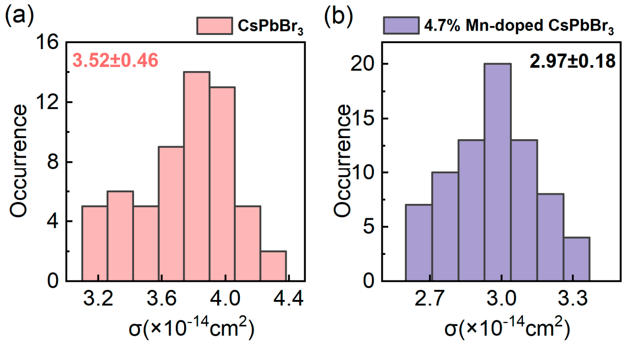

Figure S4: (a, b) Histograms of the absorption cross-sections (σ) of undoped and 4.7% Mn-doped $CsPbBr_3$ perovskite QDs.

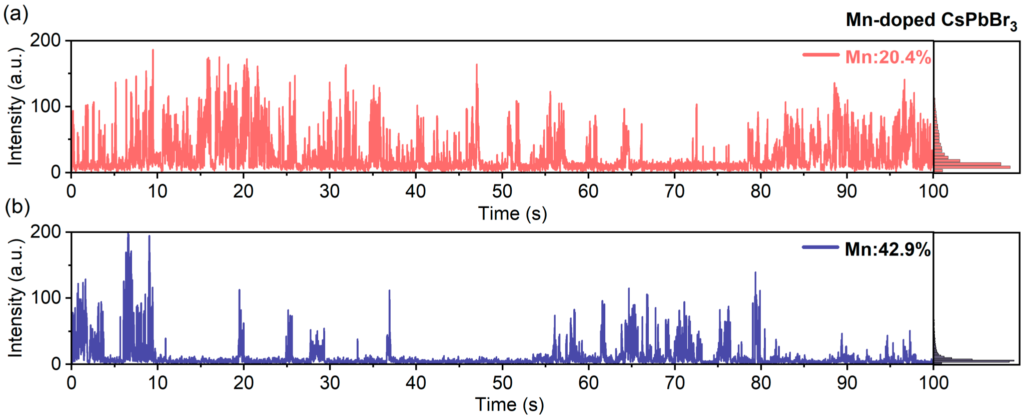

Figure S5: (a) A typical PL intensity time trajectory for 20.4% Mn-doped $CsPbBr_3$ perovskite QDs. The corresponding PL intensity histogram is shown on the right panel. (b) A typical PL intensity time trajectory for 42.9% Mn-doped $CsPbBr_3$ perovskite QDs. The corresponding PL intensity histogram is presented on the right panel.

Table S1: Mass and molar ratios of $PbBr_2$, $MnBr_2$ and $ZnBr_2$ at different doping concentrations.

	**$PbBr_2$(g)**	**$MnBr_2$(g)**	**$ZnBr_2$(g)**	**Molar ratio of $PbBr_2$, $MnBr_2$ and $ZnBr_2$**
**$CsPbBr_3$**	0.075	0	0.18	1: 0: 4
**4.7% Mn-doped $CsPbBr_3$**	0.075	0.043	0.135	1: 1: 3
**20.4% Mn-doped $CsPbBr_3$**	0.075	0.086	0.09	1: 2: 2
**42.9% Mn-doped $CsPbBr_3$**	0.075	0.172	0	1: 4: 0

Table S2: Quantitative elemental scanning ICP-OES analysis of Mn-doped $CsPbBr_3$ QDs under different doping concentrations.

Element	Mass fraction (ng/mL)		
	4.7% Mn-doped $CsPbBr_3$	20.4% Mn-doped $CsPbBr_3$	42.9% Mn-doped $CsPbBr_3$
Cs	992.442	947.818	987.97
Pb	1959.048	2221.093	2271.45
Mn	25.515	151.116	453.36
Doping Concentration	4.7%	20.4%	42.9%

Table S3: A summary table of photophysical parameters for the Mn-doped QDs obtained in this work, and results from other literature used for comparison.

Doped NCs/QDs	Average Size (nm)	PL peak (nm)	FWHM of PL spectra (nm)	FWHM of Size Distribution (nm)	Lifetime (ns)	Biexciton QYs	Biexciton Auger rate (ns^{-1})	
$CsPbBr_3$	6.41±0.87	487.6	34.5	2.0	4.93±0.94	0.06±0.04	21.65±14.08	This work
4.7% Mn-doped $CsPbBr_3$	6.08±0.49	494.6	24.1	1.2	5.37±0.77	0.06±0.03	15.81±8.06	
20.4% Mn-doped $CsPbBr_3$	5.17±0.54	486.6	27.8	1.3	-	-	-	
42.9% Mn-doped $CsPbBr_3$	4.75±0.70	492.6	34.5	1.7	-	-	-	
Nd^{3+}: $CsPbBr_3$	5-10	500	26	-	-	-	-	ref.[1]
Mn^{2+}: $(C_4H_9NH_3)_2PbBr_4$	-	600	80	-	-	-	-	ref.[2]
Al^{3+}: $CsPbBr_3$	11.87	465	15	6.8	13.6	-	-	ref.[3]

References

1. Duan, W.; Hu, L.; Zhao, W.; Zhang, X. Rare-earth ion-doped perovskite quantum dots: synthesis and optoelectronic properties. *J. Mater. Sci. Mater. Electron.* **2022**, *33*, 19019-19025.
2. Biswas, A.; Bakthavatsalam, R.; Kundu, J. Efficient exciton to dopant energy transfer in Mn^{2+}-doped $(C_4H_9NH_3)_2PbBr_4$ two-dimensional (2D) layered perovskites. *Chem. Mater.* **2017**, *29*, 7816-7825.
3. Zeng, F.; Tan, Y.; Hu, W.; Tang, X.; Zhang, X.; Yin, H. A facile strategy to synthesize high colour purity blue luminescence aluminium-doped $CsPbBr_3$ perovskite quantum dots. *J. Lumin.* **2022**, *245*, 118788.
